# MEASURING AGE-FRIENDLINESS OF TRANSPORTATION, MOBILITY COMMUNITY CHARACTERISTICS: A SCOPING REVIEW

**DOI:** 10.1093/geroni/igac059.2740

**Published:** 2022-12-20

**Authors:** Kellia Hansmann, David Deemer, Carolyn McAndrews, Stephanie Robert

**Affiliations:** William S Middleton Memorial Veterans Hospital, Madison, Wisconsin, United States; University of Wisconsin School of Medicine and Public Health, Madison, Wisconsin, United States; University of Wisconsin Madison, Madison, Wisconsin, United States; University of Wisconsin Madison, Madison, Wisconsin, United States

## Abstract

Age-friendly community initiatives seek to identify and develop infrastructure and services that promote active aging. Inclusive characteristics of transportation and outdoor spaces can improve everyone’s mobility around their communities but are particularly important to adults who are transitioning to non-driving. In this study, we aimed to identify and describe age-friendly community indicators that evaluate characteristics most associated with community-level mobility, specifically transportation and outdoor spaces. We conducted a scoping review of peer-reviewed and gray literature to find records describing the development of age-friendly community indicators and then to identify indices with measures of transportation and outdoor spaces resources. We searched PubMed, Scopus, Ebsco, and Web of Science in August 2022 for literature published since 2005. Our search resulted in 1,012 unique records; screening for relevance by title and abstract resulted in 33 records that received full text reviews. We identified 9 final indicators for inclusion and reviewed records to describe the source of the indices, development methodology, and specific indicators used to measure transportation and outdoor spaces. Included indicators were sponsored by governmental or non-profit public health organizations (n=4) and academic researchers (n=5), including one academic partnership with a for-profit insurance company. Most indices (n=6) described measures drawn from subjective data collected during community assessments. Age-friendly indicators were not developed specifically for investigating older adults’ transition to non-driving, a missing focus that represents a significant gap. Aligning indicators with this common life transition will facilitate research into the interventions promoting mobility at the community level for all.

